# Supplementary material for: Effect of thermal ballast loading on temperature stability of domestic refrigerators used for vaccine storage
Source: PLoS One. 2020 Jul 8;15(7):e0235777. doi: 10.1371/journal.pone.0235777 (PMC7343171; doi:10.1371/journal.pone.0235777)
Supplement: S1 Fig — (DOCX) [file pone.0235777.s001.docx]

**
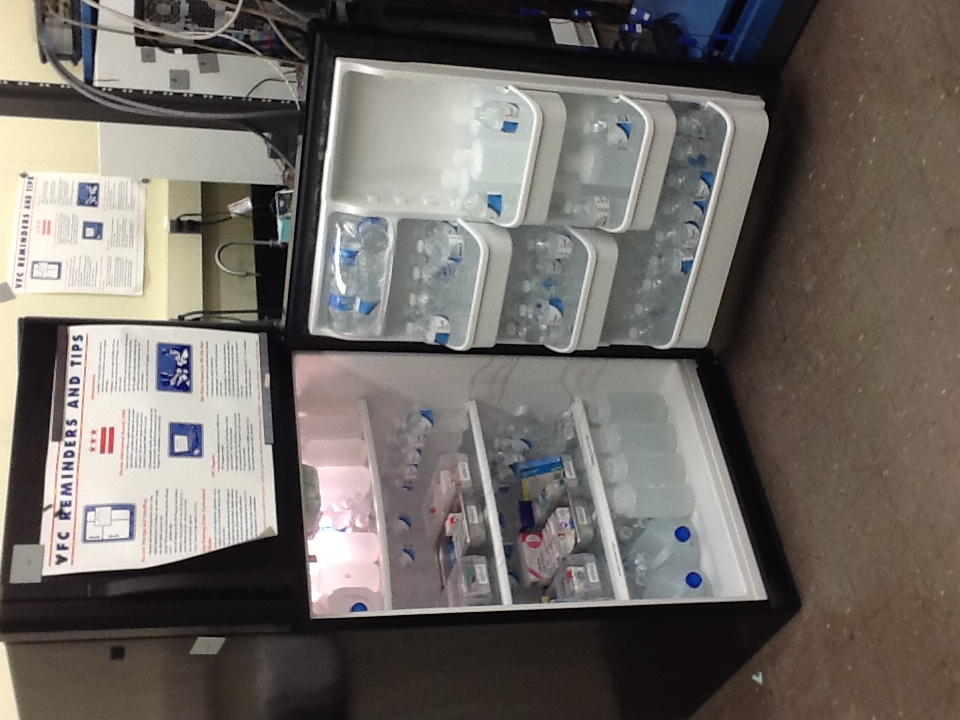
Fig 1. Combination refrigerator with 25 % ballast load.**


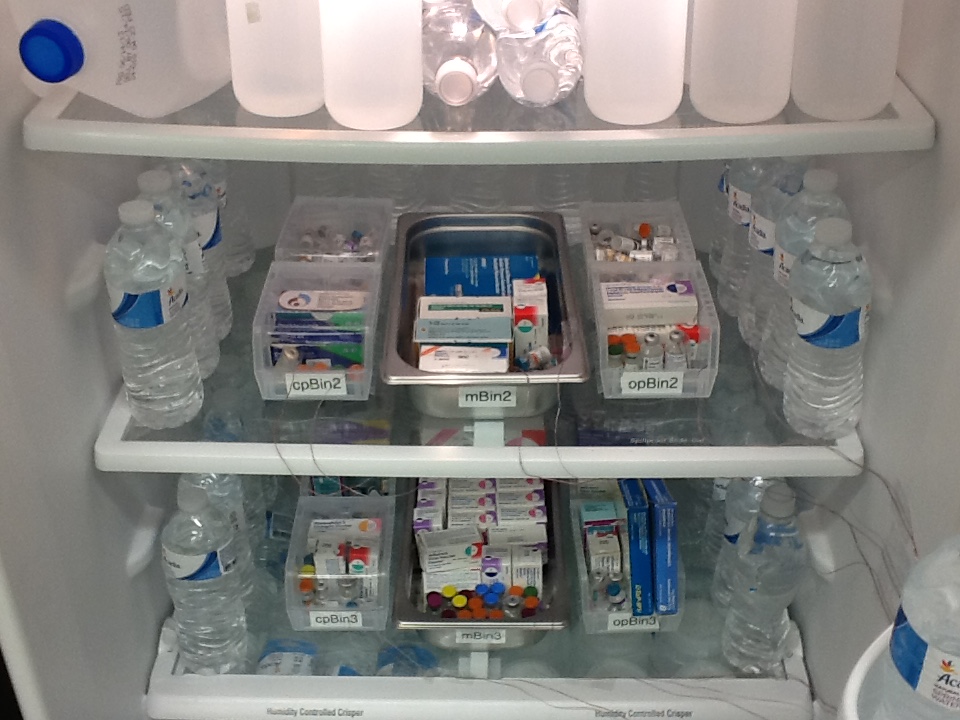


**Fig 2. Combination refrigerator with 25 % ballast load.**


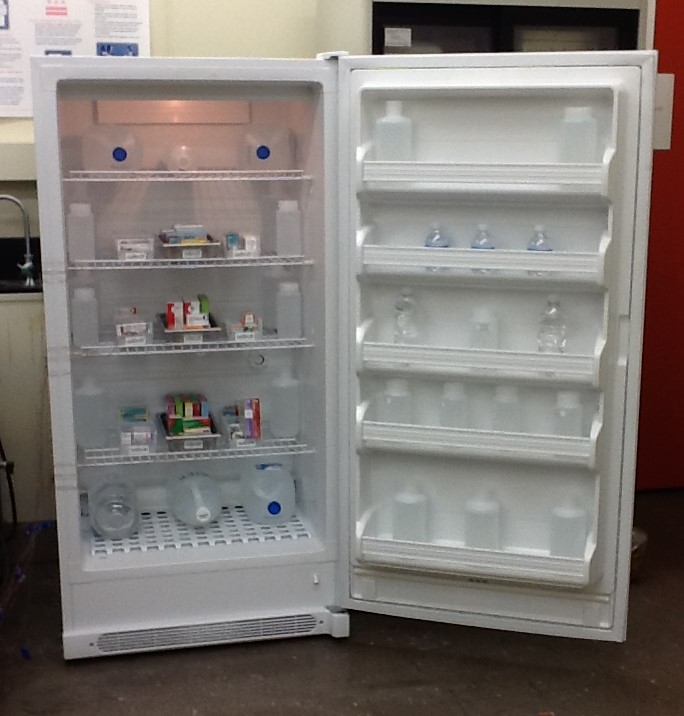


**Fig 3. Standalone refrigerator with 8% ballast load.**
