# Supplementary material for: Effect of thermal ballast loading on temperature stability of domestic refrigerators used for vaccine storage
Source: PLoS One. 2020 Jul 8;15(7):e0235777. doi: 10.1371/journal.pone.0235777 (PMC7343171; doi:10.1371/journal.pone.0235777)
Supplement: S2 Fig — (PDF) [file pone.0235777.s002.pdf]

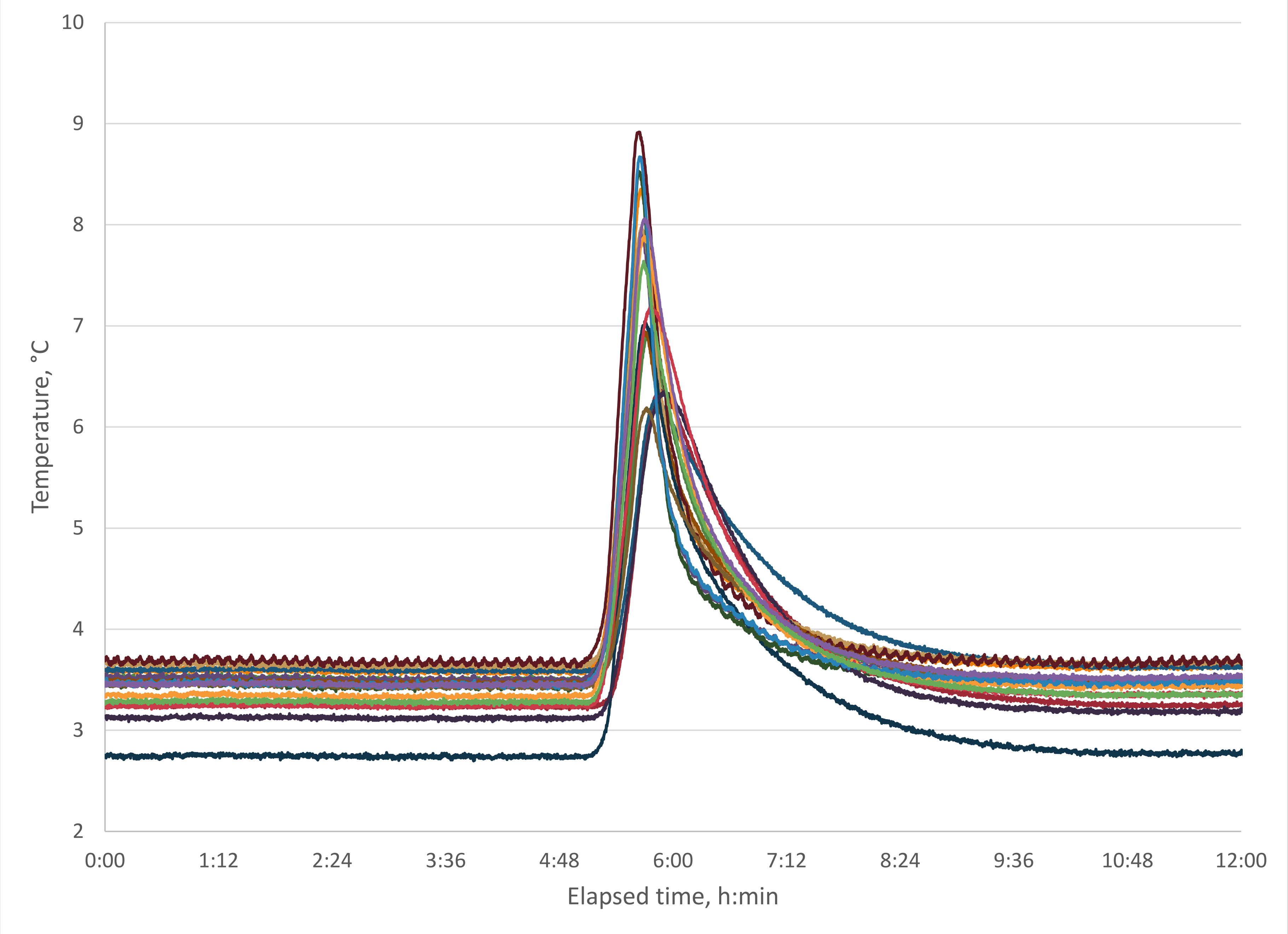

S2 Fig. Temperature response of seventeen monitored vaccine vials to defrost cycle activation in standalone refrigerator, with 0 % ballast load.
