# Supplementary material for: Effect of thermal ballast loading on temperature stability of domestic refrigerators used for vaccine storage
Source: PLoS One. 2020 Jul 8;15(7):e0235777. doi: 10.1371/journal.pone.0235777 (PMC7343171; doi:10.1371/journal.pone.0235777)
Supplement: S1 Appendix — (PDF) [file pone.0235777.s011.pdf]

S1 Appendix. Comparison of temperature response by monitored vaccines stored in different types of trays.

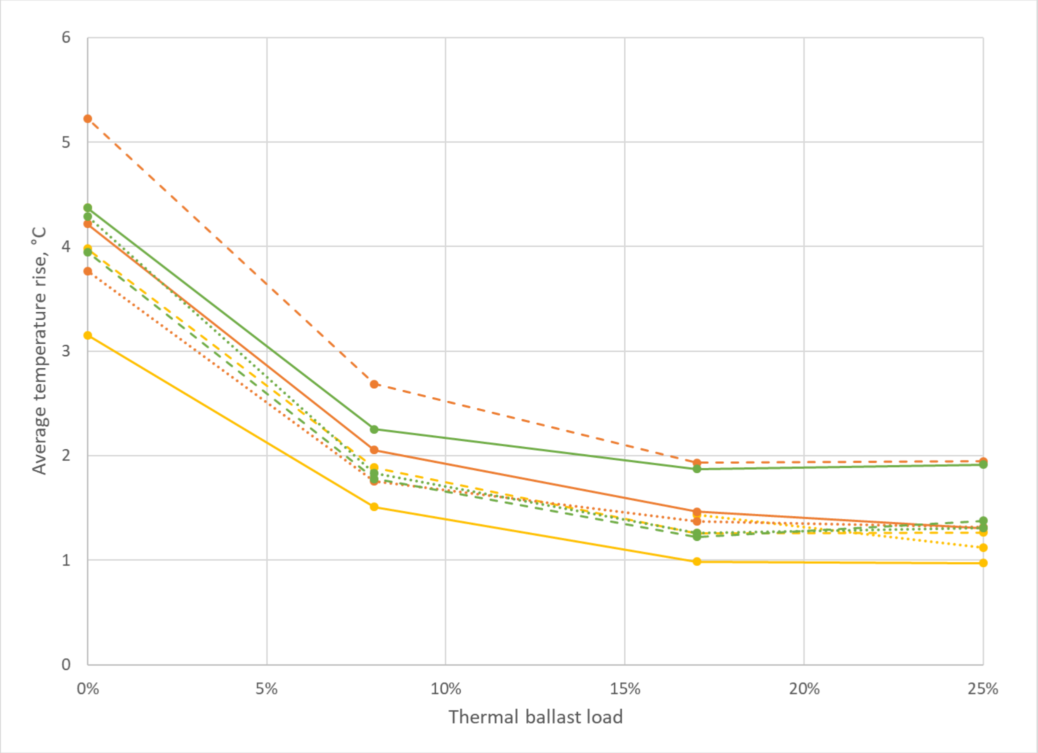

Fig 1. Defrost cycle temperature response, by tray type, in standalone refrigerator.

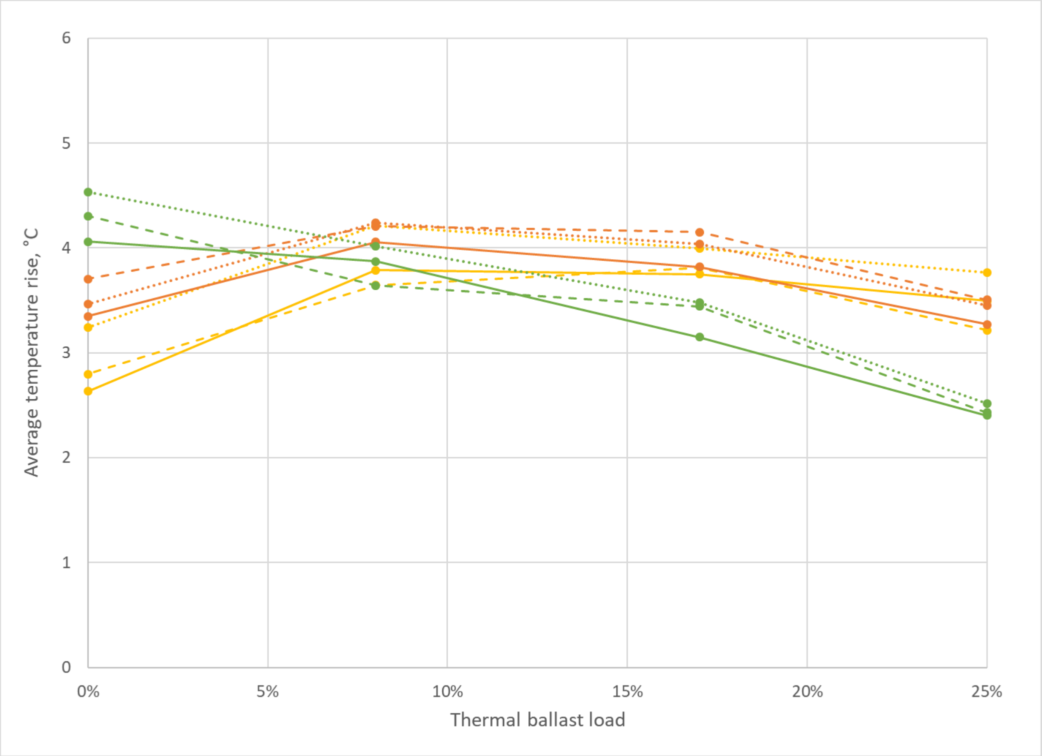

Fig 2. Repeated door opening temperature response, by tray type, in standalone refrigerator.

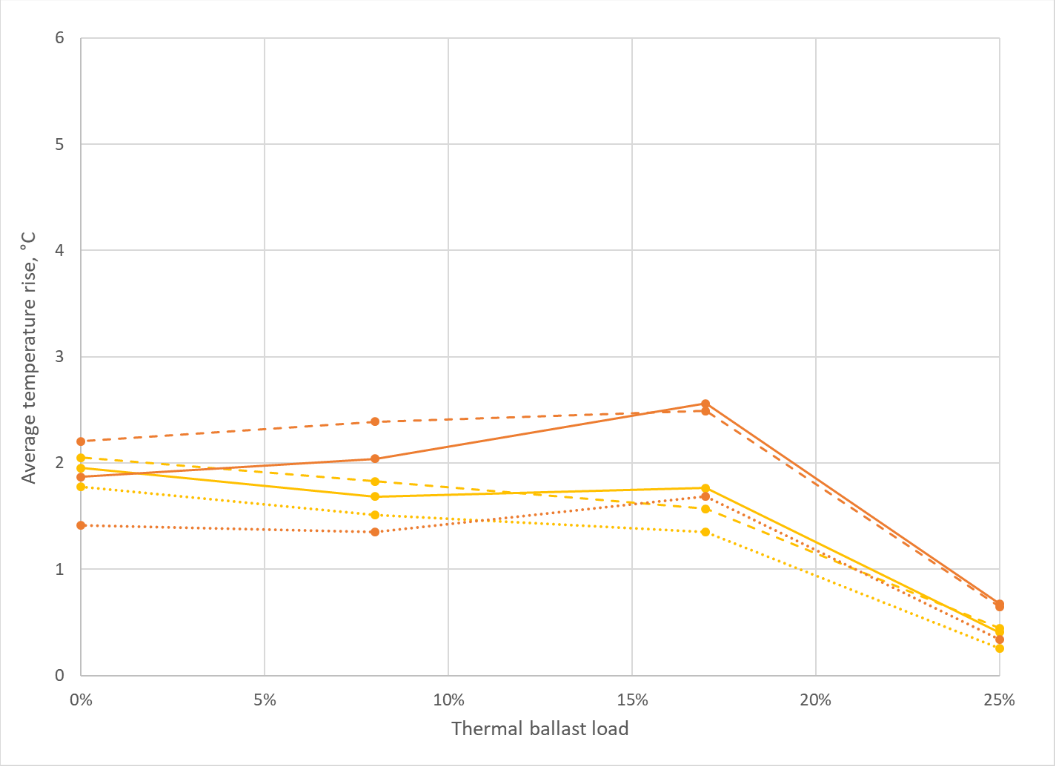

Fig 3. Defrost cycle temperature response, by tray type, in combination refrigerator/freezer.

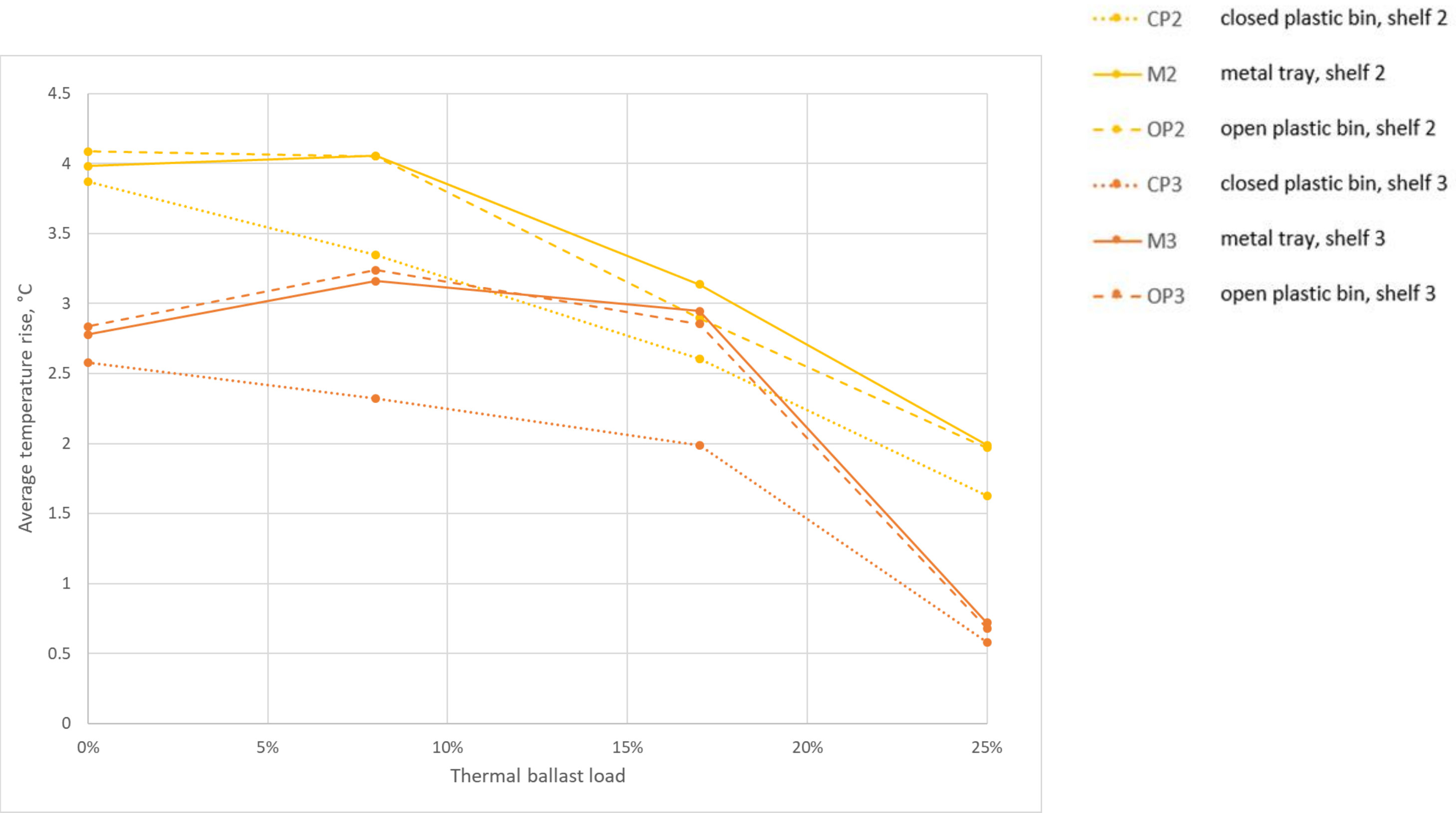

Fig 4. Repeated door opening temperature response, by tray type, in combination refrigerator/freezer.
