# Supplementary material for: Effect of thermal ballast loading on temperature stability of domestic refrigerators used for vaccine storage
Source: PLoS One. 2020 Jul 8;15(7):e0235777. doi: 10.1371/journal.pone.0235777 (PMC7343171; doi:10.1371/journal.pone.0235777)

S2 Appendix. Comparison of door opening temperature response by boxed and unboxed vaccine, at 0 % and 25 % thermal ballast loads.

Door opened for 30 seconds every 5 minutes for 2 hours

Blue = 0 % ballast load

Red = 25 % ballast load

Dashed = Unboxed Vaccine

Solid = Boxed Vaccine

Combination Refrigerator/Freezer (Dual Zone)

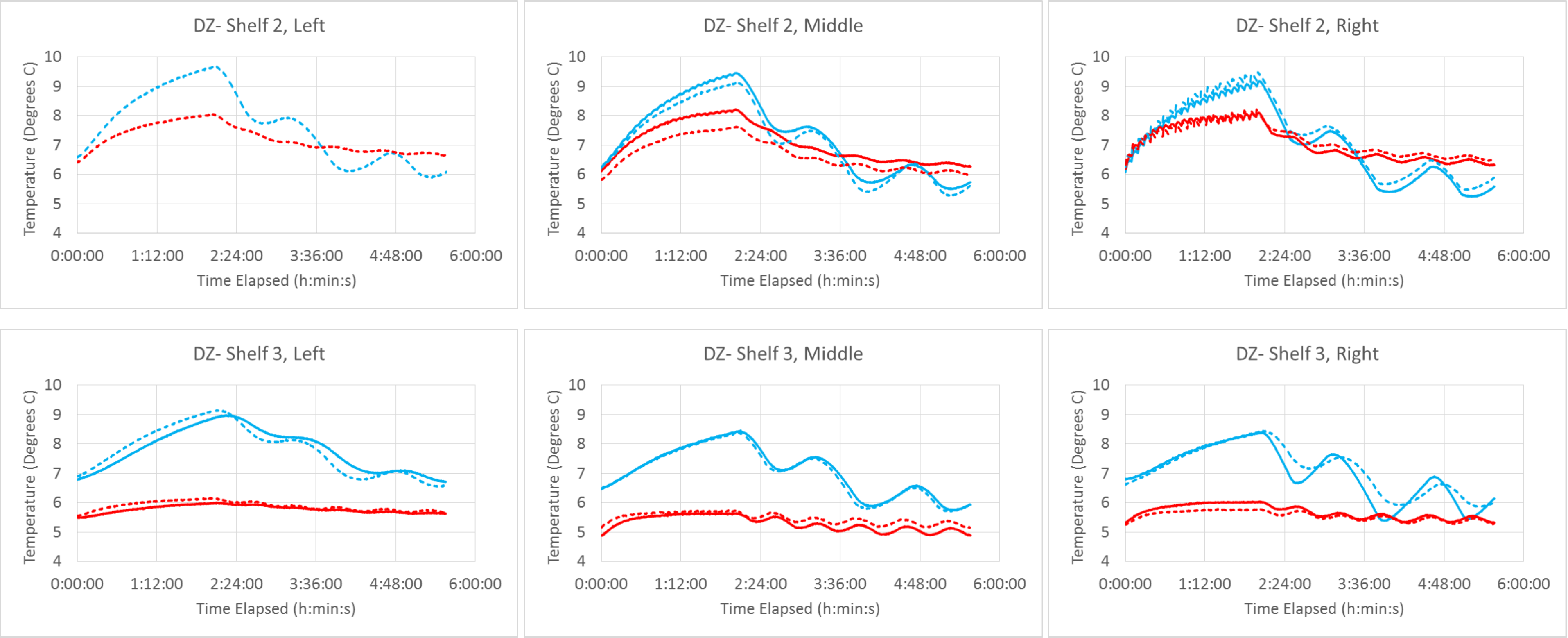

Standalone (Freezerless) Refrigerator

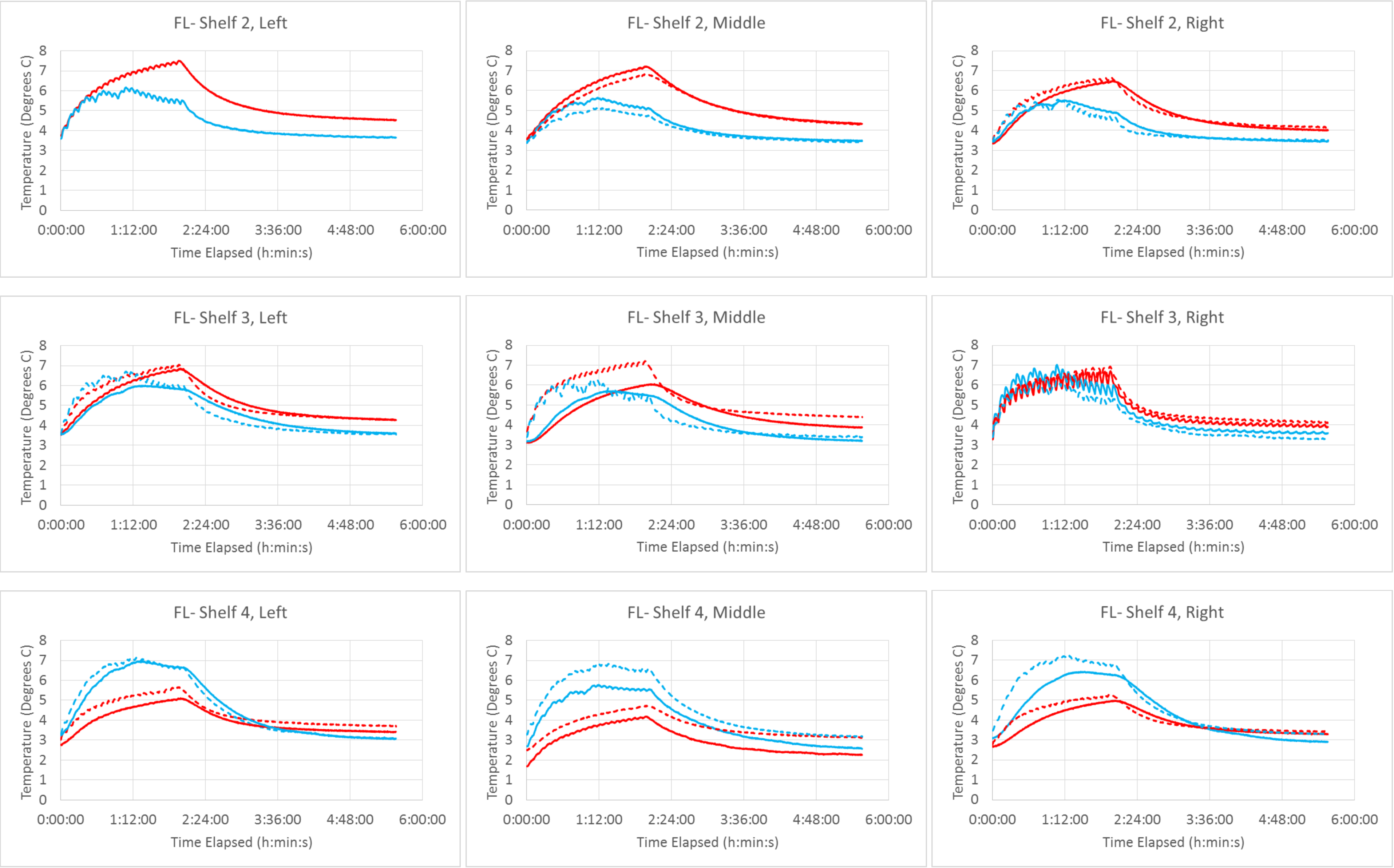

Supplement: S2 Appendix — (PDF) [file pone.0235777.s012.pdf]
