# Supplementary material for: Effect of thermal ballast loading on temperature stability of domestic refrigerators used for vaccine storage
Source: PLoS One. 2020 Jul 8;15(7):e0235777. doi: 10.1371/journal.pone.0235777 (PMC7343171; doi:10.1371/journal.pone.0235777)
Supplement: Attachment — Submitted filename: Response to Reviewers.docx [file pone.0235777.s013.docx]

Manuscript PONE-D-20-03640

Response to Reviewers

Dr. Dai-Viet N. Vo,

Thank you very much for giving us the opportunity to submit a revised draft of the manuscript, “Effect of thermal ballast loading on temperature stability of domestic refrigerators used for vaccine storage.” We appreciate the time and effort that you and the reviewers dedicated to providing feedback on our manuscript and are grateful for the insightful comments on and valuable improvements to our paper. We have incorporated most of the suggestions made by the reviewers. These changes are reflected in the track changes version of the manuscript, and a point-by-point response to each of the reviewers’ comments is provided below in **purple** text.

Please ensure that your manuscript meets PLOS ONE's style requirements, including those for file naming. The PLOS ONE style templates can be found at

<http://www.journals.plos.org/plosone/s/file?id=wjVg/PLOSOne_formatting_sample_main_body.pdf> and <http://www.journals.plos.org/plosone/s/file?id=ba62/PLOSOne_formatting_sample_title_authors_affiliations.pdf>

Minor revisions to title page to match PLOS ONE template.

Formatting revisions, including boldfaced figure titles, changed section titles to sentence case, paragraph indentation.

2. We note that you have included the phrase “data not shown” in your manuscript. Unfortunately, this does not meet our data sharing requirements. PLOS does not permit references to inaccessible data. We require that authors provide all relevant data within the paper, Supporting Information files, or in an acceptable, public repository. Please add a citation to support this phrase or upload the data that corresponds with these findings to a stable repository (such as Figshare or Dryad) and provide and URLs, DOIs, or accession numbers that may be used to access these data. Or, if the data are not a core part of the research being presented in your study, we ask that you remove the phrase that refers to these data.

Thank you for catching this. The phrase “data not shown” was mistakenly left in the text from a prior draft, and has now been removed. The data supporting the claim is included in the Supporting information files, and in text citations have been added to this paragraph.

3. Thank you for stating the following in the Competing Interests section:

"I have read the journal's policy and the authors of this manuscript have the following competing interests:

This study was partially funded by an Interagency Agreement with Centers for Disease Control and Prevention (CDC) and the authors' institution. As part of this agreement, MC provides technical guidance and research support to the CDC's Vaccines for Children program. Additionally, MC is a member of the NSF Joint Committee for Vaccine Storage, a consensus body responsible for creating and maintaining an ANSI standard for purpose-built vaccine storage equipment.".

i) Please confirm that this does not alter your adherence to all PLOS ONE policies on sharing data and materials, by including the following statement: "This does not alter our adherence to PLOS ONE policies on sharing data and materials.” (as detailed online in our guide for authors [http://journals.plos.org/plosone/s/competing-interests](https://gcc01.safelinks.protection.outlook.com/?url=http%3A%2F%2Fjournals.plos.org%2Fplosone%2Fs%2Fcompeting-interests&data=02%7C01%7Cmichal.chojnacky%40nist.gov%7C3992c0a76cb1424ff5bb08d7c4f98cda%7C2ab5d82fd8fa4797a93e054655c61dec%7C1%7C1%7C637194448399755089&sdata=%2FNmaSzpQarPhBooZFUHInu5cdOOfwwcSs%2FbPh3GPmwo%3D&reserved=0)). If there are restrictions on sharing of data and/or materials, please state these. Please note that we cannot proceed with consideration of your article until this information has been declared.

We confirm that the roles listed in our competing interest statement do not alter our adherence to the PLOS ONE policies on data sharing and materials.

ii) Please include your updated Competing Interests statement in your cover letter; we will change the online submission form on your behalf.

The updated competing interests statement has been added to the cover letter.

Please know it is PLOS ONE policy for corresponding authors to declare, on behalf of all authors, all potential competing interests for the purposes of transparency. PLOS defines a competing interest as anything that interferes with, or could reasonably be perceived as interfering with, the full and objective presentation, peer review, editorial decision-making, or publication of research or non-research articles submitted to one of the journals. Competing interests can be financial or non-financial, professional, or personal. Competing interests can arise in relationship to an organization or another person. Please follow this link to our website for more details on competing interests: [http://journals.plos.org/plosone/s/competing-interests](https://gcc01.safelinks.protection.outlook.com/?url=http%3A%2F%2Fjournals.plos.org%2Fplosone%2Fs%2Fcompeting-interests&data=02%7C01%7Cmichal.chojnacky%40nist.gov%7C3992c0a76cb1424ff5bb08d7c4f98cda%7C2ab5d82fd8fa4797a93e054655c61dec%7C1%7C1%7C637194448399765056&sdata=IvMyb%2B4Udc0bTxWWmkPR%2FmKRcVKbFply9MjAaHq7cqg%3D&reserved=0)

4. Please include captions for your Supporting Information files at the end of your manuscript, and update any in-text citations to match accordingly. Please see our Supporting Information guidelines for more information: [http://journals.plos.org/plosone/s/supporting-information](https://gcc01.safelinks.protection.outlook.com/?url=http%3A%2F%2Fjournals.plos.org%2Fplosone%2Fs%2Fsupporting-information&data=02%7C01%7Cmichal.chojnacky%40nist.gov%7C3992c0a76cb1424ff5bb08d7c4f98cda%7C2ab5d82fd8fa4797a93e054655c61dec%7C1%7C1%7C637194448399765056&sdata=BDv40IO6kD7zoC13ZuAdAkR0tbHTPLQVOEcgEPv%2BhdA%3D&reserved=0).

Supporting information captions have been added.

[Note: HTML markup is below. Please do not edit.]

Reviewers' comments:

Reviewer's Responses to Questions

**Comments to the Author**

1. Is the manuscript technically sound, and do the data support the conclusions?

The manuscript must describe a technically sound piece of scientific research with data that supports the conclusions. Experiments must have been conducted rigorously, with appropriate controls, replication, and sample sizes. The conclusions must be drawn appropriately based on the data presented.

Reviewer #1: Yes

Reviewer #2: Yes

2. Has the statistical analysis been performed appropriately and rigorously?

Reviewer #1: Yes

Reviewer #2: Yes

3. Have the authors made all data underlying the findings in their manuscript fully available?

The [PLOS Data policy](https://gcc01.safelinks.protection.outlook.com/?url=http%3A%2F%2Fwww.plosone.org%2Fstatic%2Fpolicies.action%23sharing&data=02%7C01%7Cmichal.chojnacky%40nist.gov%7C3992c0a76cb1424ff5bb08d7c4f98cda%7C2ab5d82fd8fa4797a93e054655c61dec%7C1%7C1%7C637194448399765056&sdata=dqTiCIEeT9S7UGqGFoft1RGVR%2BHRHXMKzN4vygNIJQo%3D&reserved=0) requires authors to make all data underlying the findings described in their manuscript fully available without restriction, with rare exception (please refer to the Data Availability Statement in the manuscript PDF file). The data should be provided as part of the manuscript or its supporting information, or deposited to a public repository. For example, in addition to summary statistics, the data points behind means, medians and variance measures should be available. If there are restrictions on publicly sharing data—e.g. participant privacy or use of data from a third party—those must be specified.

Reviewer #1: Yes

Reviewer #2: Yes

4. Is the manuscript presented in an intelligible fashion and written in standard English?

PLOS ONE does not copyedit accepted manuscripts, so the language in submitted articles must be clear, correct, and unambiguous. Any typographical or grammatical errors should be corrected at revision, so please note any specific errors here.

Reviewer #1: Yes

Reviewer #2: Yes

5. Review Comments to the Author

Please use the space provided to explain your answers to the questions above. You may also include additional comments for the author, including concerns about dual publication, research ethics, or publication ethics. (Please upload your review as an attachment if it exceeds 20,000 characters)

Reviewer #1: The study entitled “Effect of thermal ballast loading on temperature stability of domestic refrigerators used for vaccine storage” by Michal Chojnacky et al. investigated the effect of thermal ballast loadings on the temperature stability of vaccine stored. In general, the manuscript is well written and organized, the language is excellent so it is easy to follow. The study is every interesting because the authors compared their results on two kinds of refrigerators involving combination and standalone. Moreover, many factors (defrost cycle, door open, power outage, storage containers, glass shelves vs. wire shelves) have been studied carefully. The results seem reliable with repeatable runs. The prospective of this study is promising and meaningful practically since it can be applied for isolated regions with a deficiency of cooling instruments or electricity. With these reasons, the reviewer is in favor of acceptance for publication after the manuscript is revised along with the following minor comments.
1. The abstract of this manuscript seems quiet long and it should also be appropriately adhered according to the journal’s guide, please check and shorten it. By the way, please check the table of reference contents.

Thank you for nothing this. The abstract has been shortened to less than 300 words, per PLOS One guidelines.

2. In the conclusion, I think that you should correct first two sentences because they don’t come from your results. The conclusion should be a summary in the method, the most highlighted results, what the findings attained, etc. Please insert some numeric data to more consolidate the significance of this study.

Thank you for the suggestion. The conclusion has been updated according to these recommendations.

3. In Figs. 1-3, the name and position of the important materials (thermos ballast, vaccines, etc.) should be clarified.

We agree that this could be made clearer. Captions for Figs. 1-3 have been updated acordingly, and Fig. 2 has been recolored for clarity.

In addition, each type of vaccine is characterized by various conditions in critical temperature, light-exposure intensity, expiration date, etc. The same case is also applied for refrigerator. Please list these properties of those vaccines/refrigerator in your experiments to make sure that the further studies can be repeated to verify.

Thank you for pointing this out. We have added a descriptive sentence regarding the types and formats of vaccines used to fill the refrigerators in this study. Additional refrigerator details have also been added.

Although we agree that specific vaccine stability characteristics are important considerations for vaccine storage applications, this falls outside the scope of the present work. The focus of this manuscript is on the thermal performance of refrigerators used with thermal ballast loading techniques, and so specific data on vaccines used in the study (like expiration dates or light sensitivity) was not tracked, nor were vaccines measured for potency or light degradation at any point during the study. Rather, a selection of vaccines provided by the CDC’s Vaccine for Children program was used as a representative sample of the sort of product a typical U.S. childhood vaccine provider might stock in a refrigerator. The purpose of the vaccines in this case was merely to represent the thermal mass and behavior of the packaging, vials, and contained liquid when subjected to typical refrigerator use conditions such as door opening and power outages.

4. In Fig. 6, there are increasing trends in viable storage time along with the percentage of ballast loading for both combination and standalone refrigerators. You are highly recommended to increase ballast loadings (e.g. 30%, 35%, 40%, or more) to track the result and detect the optimum ballast loadings.
Thank you for this comment. We agree that adding more ballast materials would likely extend the viable storage time even further. However, we do not believe thermal ballast loads greater than 25 % are practical or feasible for use by vaccine providers, the intended audience for this work. The calculated ballast load percentages are based on the manufacturer-specified refrigerator capacities – however, the “usable storage volume” in a domestic refrigerator ends up being much less than this due to positioning of shelves and size/format of stored products. We have included a photo of the 2 5% ballast load in the supplementary files (S9 Figures) for reference. As you may observe, the refrigerator is quite heavily loaded even at this level, and there is not much space left for actual storage of vaccine. While we expect to get even “better” results at a 30 % or 40 % load, we don’t think this data would be useful for providers in the field, since it would so drastically reduce their usable storage volume. For this reason, we suggest a 10 % - 15 % thermal ballast load as a good balance between providing increased protection during power loss events, while still allowing for enough vaccine storage space.

Reviewer #2: This work investigated the effect of thermal ballast loading on temperature stability of domestic refrigerators used for vaccine storage. The work is interesting with relevant application to the healthcare area. The discussion is well-written; nonetheless, certain parts especially the method section are unclear. Therefore, I would like to suggest a minor revision for this work.

1) Introduction
a) Please specify what kind of “certain” operating conditions.
“However, under certain operating conditions, domestic refrigerators can expose vaccines to temperatures outside the permissible storage range (8–13).”
This sentence has been revised to better reflect the findings of the referenced articles.

2) Refrigerator Selection
a) How do the authors confirm the variation of shelve type do not affect the performance of refrigerator?
“The combination unit had three glass storage shelves inside its refrigerated compartment, and the standalone unit had four interior wire shelves.”
This possibility was explored in the case study described in the Glass Shelves vs. Wire Shelves section, in which glass shelves were added to the standalone unit (which originally came with wire shelves). A sentence was added to the Refrigerator Selection section directing interested readers to this section.

b) More specifications of refrigerators should be provided in Table 1. For instance, power consumption and etc.

Additional refrigerator details have been added.

Refrigerator power consumption falls outside the scope of this work, which was focused on temperature stability of stored vaccines. As a result, average power consumption was not measured directly, nor is this data available from the EPA or manufacturer databases, as these models were discontinued some time ago. However, interested readers may calculate an estimate based on the provided electrical ratings.

c) The model of refrigerators should be mentioned.
Refrigerator model numbers have been added.

3) Temperature Monitoring
a) Any specific reason for using different number of thermocouples to monitor vaccines from different refrigerators?
“A total of twenty-nine Type T thermocouples were used to monitor vaccines in the two refrigerators, with twelve monitored vaccines in the combination unit, and seventeen monitored vaccines in the standalone unit.”

Six thermocouples were used to monitor vaccine vials on each shelf used for vaccine storage in this study, as shown in Fig. 2. The standalone refrigerator had 4 shelves in total, and the combination refrigerator/freezer unit had 3 shelves in its refrigerator compartment. In both units, the top shelf was reserved for storing water bottles, so no vaccines or sensors were placed on that level. As a result, we used 12 thermocouples in the combination unit to cover the remaining two shelves, and 18 thermocouples to cover the remaining three shelves in the standalone unit. One of the thermocouples in the standalone unit broke during testing and had to be eliminated, leaving 17 thermocouples.

b) What is type A and type B temperature measurement systems? Are them refers to thermocouple? If yes, why use two different thermocouples? Confusing as they are not mentioned in the text.
Only one type of thermocouple was used in the study. The table in question gives a breakdown of the measurement uncertainties associated with the thermocouples and the other components of the measurement system (the reference junction bath and multimeter).

Type A and B refer the type of measurement uncertainty. Type A uncertainties are derived from statistical methods, and Type B uncertainties are based on things like previous measurement data or manufacturer's specifications. We added “uncertainties” to the table to clarify this point, and included a reference which describes the methods used to calculate these uncertainties in detail.

4) Thermal Ballast
a) Are the large bottles and jugs also made from plastic? Different matters might have different heat conductivity.
Yes, the larger bottles and jugs are also made of plastic, and this has been added to the sentence in question.

b) The formula of temperature stability should be included in the methodology section instead of results & discussion section.
Thank you for pointing this out. We agree it makes more sense to include this in the Methods discussion. An Evaluation Methods subsection has been added to the Methods section, and the evaluation method and terminology is now defined here.

5) Test Protocol
a) The set temperature should be specified in the text.

The set point temperature was not explicitly set or calibrated, but the refrigerator temperature control dials were kept fixed on a standard midpoint setting. This has been added to the Refrigerator Selection subsection. We accounted for variation in set point via our analysis method, in which we look at a temperature shifts (e.g. $\overline{\Delta T}>3 )$ in response to each use trial, rather than absolute temperatures.

6) Defrost cycle
a) Transient profile of temperature should be provided to indicate any fluctuation over time.
An example of the transient temperature profile during a defrost cycle has been referenced and included as a supporting file, S1 Fig.

b) Please specify how many % of reduced temperature fluctuation inside bracket.
“…while the 8 % and 17 % ballast loads had little effect on the defrost cycle temperature response.”
We have changed the wording to clarify - “However, smaller ballast loads had little effect on the defrost cycle temperature response. As shown in Fig. 2, temperature cycling was nearly identical for the 0 %, 8 %, and 17 % loads.”

7) Repeated Door Opening
a) Redundant discussion. The way of evaluation should be declared in methodology rather than discussion.
“…with Δ > 3 ° indicative of a potential excursion condition.”
This is an excellent suggestion. We have added an Evaluation Methods sub-section to Methods section. The evaluation method is now described here, and redundant language has been eliminated from later Results discussion.

8) Door Open for One Hour
a) Similar to comment #6a.
“As before, we examined the average temperature change of the monitored vaccine vials, Δ, to assess temperature stability during the trial. We define “viable vaccine storage time” as the duration of time in which vaccines were maintained within 3 °C of their initial, pre-door opening storage temperature (Δ < 3 °).”
We have added an Evaluation Methods sub-section to Methods section. The evaluation method is now described here, and redundant language has been eliminated from later Results discussion.

b) I suggest the authors to provide the data as supplementary data.
“In this case, vaccines remained within a viable storage temperature range for an average of 22 minutes (data not shown).”

Thank you for catching this. The appropriate supplementary files (S5 Table and S6 Table) are now referenced, and the erroneous phrase “data not shown” has been removed.

9) Power Outage
a) Similar to comment #6a.
“To determine the impact of thermal ballast load on refrigerated vaccine temperature stability during a power outage event, we examined “viable vaccine storage time” during simulated outage tests performed at different ballast load percentages. As before, viable vaccine storage time is defined as the duration of time during which vaccine temperatures were maintained within 3 °C of their initial, pre-outage storage temperature (Δ < 3 °). In this framework, a temperature rise of more than 3 °C is equivalent to the start of a temperature excursion, and the end of viable vaccine storage time.”
We have added an Evaluation Methods sub-section to Methods section. The evaluation method is now described here, and redundant language has been eliminated from later Results discussion.

b) How the Pearson correlation coefficient being calculated? It should be included under the methodology section.
“Pearson correlation coefficient, r, was calculated to assess the strength of the relationship between added thermal ballast and duration of viable vaccine storage time during an outage, for each tested unit.”

“…n is the total number of observations (temperature sensors multiplied by number of trials), and p is the probability that the null hypothesis (added thermal ballast is not associated with an increase in viable storage time) is true”
The Pearson correlation coefficient calculation method is now described and the formula has been included in the Evaluation Methods sub-section.

10) Storage Containers
a) How trivial it is? Please provide some numerical values or graph?
“Still, our results suggest that the selection of a storage container (metal, plastic, open vs. closed) is trivial in terms of its impact on maintaining stored vaccine temperatures.”

Thank you for bringing this to our attention. This discussion has been revised and a supplementary file (S2 Appendix) has been added, showing the observed variation due to storage container type.

11) Glass Shelves vs. Wire Shelves
a) The methodology of this section should be included under the methodology section.
“Shelf Material Case Study” subsection has been added to Materials and Methods.

b) Please provide the specification of the glass sheet that used to produce glass shelves.

Additional specifications and identifying information about the glass sheets have been added to the Shelf Material Case Study subsection.

12) Discussions
a) It is well-written. Well done.

Thank you!

13) Conclusion
a) The conclusion should be more specific in accordance to the current findings. Please support your recommendation/suggestion with facts like data.

 Thank you for the suggestion. The conclusion has been updated according to your recommendations.

6. PLOS authors have the option to publish the peer review history of their article ([what does this mean?](https://gcc01.safelinks.protection.outlook.com/?url=https%3A%2F%2Fjournals.plos.org%2Fplosone%2Fs%2Feditorial-and-peer-review-process%23loc-peer-review-history&data=02%7C01%7Cmichal.chojnacky%40nist.gov%7C3992c0a76cb1424ff5bb08d7c4f98cda%7C2ab5d82fd8fa4797a93e054655c61dec%7C1%7C1%7C637194448399775008&sdata=lXltnPEi%2Flg%2BJ3gJXtPpJ2H6t3u17lxV4cceHd4S5g8%3D&reserved=0)). If published, this will include your full peer review and any attached files.


If you choose “no”, your identity will remain anonymous but your review may still be made public.


**Do you want your identity to be public for this peer review?** For information about this choice, including consent withdrawal, please see our [Privacy Policy](https://gcc01.safelinks.protection.outlook.com/?url=https%3A%2F%2Fwww.plos.org%2Fprivacy-policy&data=02%7C01%7Cmichal.chojnacky%40nist.gov%7C3992c0a76cb1424ff5bb08d7c4f98cda%7C2ab5d82fd8fa4797a93e054655c61dec%7C1%7C1%7C637194448399775008&sdata=T3WWcroJpQP9owI4u%2FRrmyMkp9tnStoTKw7zWh3ufBM%3D&reserved=0).

Reviewer #1: No

Reviewer #2: No


[NOTE: If reviewer comments were submitted as an attachment file, they will be attached to this email and accessible via the submission site. Please log into your account, locate the manuscript record, and check for the action link "View Attachments". If this link does not appear, there are no attachment files to be viewed.]


While revising your submission, please upload your figure files to the Preflight Analysis and Conversion Engine (PACE) digital diagnostic tool, [https://pacev2.apexcovantage.com/](https://gcc01.safelinks.protection.outlook.com/?url=https%3A%2F%2Fpacev2.apexcovantage.com%2F&data=02%7C01%7Cmichal.chojnacky%40nist.gov%7C3992c0a76cb1424ff5bb08d7c4f98cda%7C2ab5d82fd8fa4797a93e054655c61dec%7C1%7C1%7C637194448399784966&sdata=RLiI0F%2Fr54p5tYic5v2Wp99mu4glUn1YSzLsCoLYcuw%3D&reserved=0). PACE helps ensure that figures meet PLOS requirements. To use PACE, you must first register as a user. Registration is free. Then, login and navigate to the UPLOAD tab, where you will find detailed instructions on how to use the tool. If you encounter any issues or have any questions when using PACE, please email us at [figures@plos.org](mailto:figures@plos.org). Please note that Supporting Information files do not need this step.
